# Waiting time for kidney transplantation based on calculated panel reactive antibodies: experience of a southern Brazilian center

**DOI:** 10.1590/2175-8239-JBN-2022-0132en

**Published:** 2023-09-25

**Authors:** Lisianara Acosta Ramos, Tiago Schiavo, Juliana Montagner, Cristiane Bundcher, Roger Kist, Valter Duro Garcia, Jorge Neumann, Elizete Keitel

**Affiliations:** 1 Universidade de Ciências da Saúde de Porto Alegre, Porto Alegre, RS, Brazil.; 2 Santa Casa de Misericórdia de Porto Alegre, Porto Alegre, RS, Brazil.

**Keywords:** Waiting Lists, Kidney Transplantation, anti-HLA antibodies, cPRA, Listas de Espera, Transplante de Rim, anticorpos anti-HLA, PRAc

## Abstract

**Introduction:**

The aim of this study was to analyze the waiting list for kidney transplantation in our hospital according to candidate’s panel reactive antibodies (cPRA) and its outcomes.

**Methods:**

One thousand six hundred forty patients who were on the waiting list between 2015 and 2019 were included. For the analysis, hazard ratios (HR) for transplant were estimated by Fine and Gray’s regression model according to panel reactivity and HR for graft loss and death after transplantation.

**Results:**

The mean age was 45.39 ± 18.22 years. Male gender was predominant (61.2%), but the proportion decreased linearly with the increase in cPRA (p < 0.001). The distribution of patients according to panels were: 0% (n = 390), 1% – 49% (n = 517), 50% – 84% (n = 269), and ≥ 85% (n = 226). Transplantation was achieved in 85.5% of the sample within a median time of 8 months (CI 95%: 6.9 – 9.1). The estimated HRs for transplantation during the follow-up were 2.84 (95% CI: 2.51 – 3.34), 2.41(95%CI: 2.07 – 2.80), and 2.45(95%CI: 2.08 – 2.90) in the cPRA range of 0%, 1%–49%, and 50%–84%, respectively, compared to cPRA ≥ 85 (p < 0.001). After transplantation, the HR for graft loss was similar in the different cPRA groups, but the HR for death (0.46 95% CI 0.24–0.89 p = 0.022) was lower in the 0% cPRA group when adjusted for age, gender, and presence of donor specific antibodies (DSA).

**Conclusion:**

Patients with cPRA below 85% are more than twice as likely to receive a kidney transplantation with a shorter waiting time. The risk of graft loss after transplantation was similar in the different cPRA groups, and the adjusted risk of death was lower in nonsensitized recipients.

## RESUMO

**Introdução:**

O objetivo foi analisar a lista de espera para transplante renal em nosso hospital segundo o painel de reatividade de anticorpos (PRAc) do candidato e seus desfechos.

**Métodos:**

Incluímos 1.640 pacientes em lista de espera entre 2015 e 2019. Para a análise, estimou-se a razão de risco (HR) para transplante pelo modelo de regressão de Fine e Gray conforme o painel de reatividade e HR para perda do enxerto e óbito após o transplante.

**Resultados:**

A idade média foi 45,39 ± 18,22 anos. Sexo masculino foi predominante (61,2%), mas a proporção diminuiu linearmente com o aumento do PRAc (p < 0,001). A distribuição de pacientes conforme os painéis foi: 0% (n = 390), 1% – 49% (n = 517), 50% – 84% (n = 269), e ≥85% (n = 226). O transplante foi realizado em 85,5% da amostra em tempo mediano de 8 meses (IC 95%: 6,9 – 9,1). As HRs estimadas para transplante durante o acompanhamento foram 2,84 (IC 95%: 2,51 – 3,34), 2,41 (IC 95%: 2,07 – 2,80) e 2,45 (IC 95%: 2,08 – 2,90) no intervalo de PRAc de 0%, 1%–49% e 50%–84%, respectivamente, comparadas com PRAc ≥ 85 (p < 0,001). Após o transplante, a HR para perda do enxerto foi semelhante nos diferentes grupos de PRAc, mas HR para óbito (0,46 IC 95% 0,24–0,89 p = 0,022) foi menor no grupo PRAc 0% quando ajustada para idade, sexo e presença de anticorpos doador específico (DSA).

**Conclusão:**

Pacientes com PRAc abaixo de 85% têm mais que o dobro de probabilidade de receber transplante renal com tempo de espera menor. Risco de perda do enxerto após o transplante foi semelhante nos diferentes grupos PRAc, e risco ajustado de óbito foi menor em receptores não sensibilizados.

## Introduction

There are about 30,000 patients waiting for a kidney transplantation in Brazil. In the Rio Grande do Sul state, the list includes between 1200 and 1300 people(ABTO)^1^. The kidney transplantation center of Santa Casa de Misericórdia de Porto Alegre contributes with about 40% of this list. The waiting time for a transplantation depends not only on available organs, but also on the candidate’s panel reactive antibodies (cPRA). The higher the cPRA, the longer the waiting list time (WLT) and the worse the expected outcomes. In the USA, 97,522 people were waiting for a kidney in March 2022, the frequency of people who received an organ according to cPRA (%) was 0: 58.6%, 0–19: 12.4%, 20–79: 17.5%, 80–97%: 5.3%, 98–100: 6.2%. The percent of those with a WLT of more than 5 years was 13.1%, 11.3%, 12.7%, 12.8%, 15.8% and 28.9%, respectively^2^. The objective of this study was to analyze the waiting list time and outcomes of our center according to cPRA and the risk of graft loss and death of transplanted recipients.

## Methods

### Population

The available data of 1,640 patients registered in our center were retrieved from the National Transplantation System (SNT) waiting list during the period from Jan 2015 to Dec 2019.

### Calculated Panel Reactive Antibodies (cPRA)

The search of antibodies was made by Luminex technology^3^. The presence of anti-HLA antibodies, considered the mean fluorescence intensity (MFI), was above 1,000. PRA was made using the frequency of antigens in a sample of 1447 HLA typed in loci A, B, C, DR, and DQ in our lab from a sample of deceased donors in Brazil, most of them from the South. The DP typing was not applied to this calculator developed in Portugal. The DP was based on the Canadian calculator (2,296 samples) that presents a similar frequency of other alleles to ours. The Canadian cPRA calculator is a component of the Canadian Transplant Registry (CTR), a web-based application used by the transplant community to estimate the percentage of Canadian deceased organ donors with whom a transplant candidate may be incompatible. This calculator uses the same formula and data as the CTR^4^. Inclusion of DQA, DPA, and DPB UA in Canadian cPRA calculations improves the accuracy of cPRA where these are relevant in allocation^5,6^. The routine in our program is to perform transplantation with cross-match by negative flow cytometry, with rare exceptions, such as urgency for transplant due to lack of access, when positive FCXM B is accepted within certain limits (Table 1).

**Table 1:** Characteristic of the patients and outcomes according to cPRA

	0% (n = 419)	1% – 49% (n = 575)	50% – 84% (n = 289)	≥85% (n = 357)	Total (n = 1640)	p-valor
	n (%)	n (%)	n (%)	n (%)	n (%)	
**Age**	46.7 ± 18.8	44.6 ± 19,5	44.7 ± 16,9	45.6 ± 16.3	45.4 ± 18.2	0.328^*^
**Gender**						
M	300 (71.6)	400 (69.6)	156 (54.0)	148 (41.5)	1004 (61.2)	**<0.001** ^#^
F	119 (28.4)	175 (30.4)	133 (46.0)	209 (58.5)	636 (38.8)	
**Outcomes**						
Active	15 (3.6)	42 (7.3)	13 (4.5)	82 (23.0)	152 (9.3)	**0.004** ^$^
Transplant	390 (93.1)	517 (89.9)	269 (93.1)	226 (63.3)	1402 (85.5)	
Death	14 (3.3)	16 (2.8)	7 (2.4)	49 (13.7)	86 (5.2)	

^*^ANOVA. Variables reported as mean ± standard deviation;

^#^Chi-square test of linear association;

^$^Chi-square adjusted by standardized adjusted residues.

### Time on List

The time on waiting list was considered the time in months since patient registration on the list until the date of transplant or death or active on list until Jul 3, 2021 (end of the study). Transplanted patients that returned to the waiting list were entered twice in time calculation as a different subject (n = 29).

### Statistical Analysis

Age is presented as mean and standard deviation and cPRA and outcomes as frequency and percentage. The association of cPRA with variables was analyzed by Chi-square of linear association and ANOVA. Transplant was the outcome of interest, death was the competitive event, and the absence of both was the censored. Also, the ROC curve was used to find the best cutoff of cPRA to predict transplant. Kaplan-Meier method was used to analyze the time until transplantation, and comparison was performed by log-rank test. Posteriorly, the follow-up time in months of transplanted recipients was calculated from the transplant date until death or graft loss or follow-up loss or end of study. Death was the outcome of interest, graft loss, the competitive event, and the absence of both, censored. The hazard ratios (HR) for transplantation of patients on the list and for death after transplantation were estimated by Fine and Gray’s regression model with 95% confidence interval (95%CI). Estimated HR were adjusted by age and gender. In the analyses of outcome after transplantations, the DSA was included as a covariate. The software SPSS (IBM SPSS Statistics for Windows, Version 25.0. Armonk, NY: IBM Corp) and the cmprsk package of R software were used for analysis. The statistical significance level adopted was 0.05. The study was approved by the local ethical committee number 3.798.838.

## Results

The sample was composed of 1,640 patients on the waiting list from 2015 to 2019. The mean age was 45.39 ± 18.22 years, without difference among cPRA groups (p = 0.328). Male was the predominant gender (61.2%) and it decreased linearly with higher cPRA (p < 0.001). The cPRA ≥85% was linearly significantly associated with the proportion of people still on the waiting list and death. The data are shown in Table 1. As can be seen in Table 2, the panels with the highest number of patients on the list were: 1% – 49% (n = 517) and 0% (n = 390). Panels with the lowest number of transplant recipients were: ≥ 85% (n = 226) and 50% – 84% (n = 269). Transplantation was achieved in 85.5% of the sample within a median of 8 months (95% CI: 6.9 – 9.1). A significant difference was found when comparing WLT by cPRA (Log Rank = 188.0 p < 0.001). The cPRA ≥ 85% had a median time until transplantation of 36 months (95% CI: 28.1 – 43.9), significantly higher than other groups. The cPRA from 50% – 84% and 1% – 49% were not different (7.0 months, 95%CI:52 – 8.8, and 6.0 months, 95% CI: 4.8 – 7.2, respectively), but significantly higher than cPRA zero (5.0 months 95%CI:6.9 – 9.1) (Table 2). The best cPRA cutoff to predict kidney transplantation was cPRA lower than 85.5%, with sensitivity of 84%, specificity 55.0%, and the area under the curve of 0.712 (95% CI 0.486–0.6123; p < 0.001) (Table 3). The analysis of the highest cPRAs showed that only 54.9% of the patients (n = 56/102) with cPRA > 99% were transplanted during follow-up time, with a median time of 47 months (95%CI 20–74). In patients with cPRA between 96 and 99%, transplantiation was achieved in 59.3% (n = 86/145) in 47 months (95%CI 35.8–58.2).

**Table 2:** Time on waiting list until transplantation by cPRA

Panel	n (%)	Median time (m)	SE	95% CI	
0%	390 (93.1)	5.0	0.5	3.9	6.1
1% – 49%	517 (89.9)	6.0	0.6	4.8	7.2
50% – 84%	269 (93.1)	7.0	0.9	5.2	8.8
≥85%	226 (63.3)	36.0	4.0	28.1	43.9
Overall	1402 (85.5)	8.0	0.5	6.9	9.1

SE: Standard error; 95%CI: 95% confidence interval; m: month.

**Table 3:** ROC Curve and time on waiting list until transplantation by cPRA

ROC analysis				Time on waiting list (month)				
AUC	95%CI	Sens.	Spec.	Cut-Off	Tx (%)	Mediam	SE	91%CI
0.712	0.672 – 0.751	84%	55%	<85%	1176 (91.7)	6.0	0.4	5.2 – 6.8
				≥85%	226 (63.3)	36.0	4.0	28.1 – 43.9

AUC: Area under curve; Sens: Sensitivity; Spec: Specificity; SE: Standard error; 95%CI: 95% Confidence interval.

The estimated HRs of kidney transplantation during follow-up were 84 (95%CI: 2.51 – 3.34), 2.41 (95%CI: 2.07 – 2.80), and 45 (95%CI: 2.08 – 90) in the cPRA range of 0%, 1%–49%, and 50%–84%, respectively, compared to cPRA ≥ 85 (p < 0.001). After adjustment by gender and age, HRs remained similar as shown in Table 4.

**Table 4:** Hazard ratio estimation of patients on waiting list for transplantation by cPRA (n = 1,640)

	p-value	HR	95%CI		p-value	HR^*^	IC95%	
**cPRA**								
0%	<0.001	2.84	2.41	3.34	<0.001	2.88	2.43	3.40
1% – 49%	<0.001	2.41	2.07	2.80	<0.001	2.43	2.09	2.84
50% – 84%	<0.001	2.45	2.08	2.90	<0.001	2.45	2.07	2.90
≥85%		1				1		
<85%	<0.001	2.53	2.21	2.91	<0.001	2.55	2.22	2.94
≥85%		1				1		

HR: Hazard ratio.

^*^Adjusted by age and gender.

The outcomes of the subgroup of patients (n = 1129) submitted to kidney transplantation with deceased donor between Jan 2015 and Dec 2019 showed no significant difference in HR for death and graft loss with different cPRA. However, after adjusted by age, gender, and presence of DSA the 0% cPRA group had lower risk of death. (Table 5).

**Table 5:** Hazard ratio estimation of death and graft loss after kidney transplantation by cPRA range (n = 1,129)

	p-value	HR	95%CI		p-value	HR^$^	95%CI	
**Outcome: death after transplantation**								
0%	0.081	0.61	0.35	1.06	0.022	0.46	0.24	0.89
1% – 49%	0.075	0.63	0.38	1.05	0.027	0.51	0.28	0.92
50% – 84%	0.157	0.65	0.36	1.18	0.089	0.58	0.31	1.09
≥85%		1				1		
**Outcome: graft loss**								
0%	0.626	1.14	0.68	1.89	0.598	1.17	0.65	2.10
1% – 49%	0.770	1.08	0.66	1.77	0.706	1.12	0.63	1.97
50% – 84%	0.857	0.95	0.54	1.67	0.911	0.97	0.54	1.74
≥85%		1				1		

HR: Hazard ratio;

^$^Adjusted by age, gender and presence of donor specific antibodies.

## Discussion

This study showed a significant greater WLT for patients with cPRA higher than 85% and specially above 95%. The cPRA cutoff to predict increase risk of staying on the waiting list defers by center depending on the sensitivity of the methods used to search for HLA antibodies and the cutoff of mean fluorescence intensity (MFI). cPRA above 85% was present in 21.7% and above 95% was present in 17.7% of waiting list patients, a higher frequency than the USA data, probably because they have implemented a new kidney allocation system (KAS) since 2014 and have increased the transplant rate in this group of patients from 2.4% to 12.3% after the first year. Luminex technology, which defines the presence of anti-HLA antibodies as MFI above 1,000, is much more sensitive to detect pre-sensitization in potential transplant recipients that is not detected by other HLA antibody detection methods, which may be another reason for higher rate of sensitized patients in our sample^7,8^.

Transplantation was performed in 85.5% of this patient sample in a median of 8 months. However, patients with cPRA ≥ 85% had a median time to transplantation of 36 months, significantly higher than the other groups and patients with cPRA above 99%, of whom only about 50% transplanted with a median of 47 months. In previous analysis prepared by Marinho et al.^9^, the average waiting time for a kidney transplant in Brazil was 1.32 years. They showed discrepancies between the regions of the country, with the South and Southeast regions being those with the shortest time intervals. However, rates were not separated by cPRA. Lim et al.^10^, who used the Australian and New Zealand Dialysis and Transplant Registry (ANZDATA), found that high pre-transplant PRA levels are associated with detrimental effects on graft outcomes. Traditionally, high PRA, re-transplant, and deceased donor grafts have been associated with an increased risk. Desensitization to prevent early antibodies-mediated rejection may be an option in some centers^11^. For patients with a cPRA > 95% and especially those approaching a 100% cPRA, the number of donors needed to have a high probability of finding an acceptable match increases exponentially. For example, to achieve a 95% probability of finding an acceptable donor, a candidate with a cPRA of 99% would need to take part in 300 donor offers, while a candidate with a cPRA of 99.5% would need 600 offers, with cPRA of 99.9% 3,000, and >99.99% would need 30,000 donor offers, stressing the need of a greater donor pool.

Our data showed that, in the absence of desensitization, patients with cPRA ≥85% had a significantly higher proportion of active status and death on the waiting list. However, highly sensitized patients with an absence of DSA and a negative flow crossmatch had similar graft survival compared to lower cPRA ranges, showing that by increasing donor pool it is possible to find a compatible donor and have a successful transplant. In this study, after adjusting for gender and age, the chance of transplantation during follow-up for patients with 0% panel compared to ≥ 85% was increased by 288%. For Lim et al.^12^, highly sensitized kidney transplant recipients with a peak PRA greater than 80% had a higher risk of rejection (at least 1.8 times compared to recipients with a peak PRA level of 0%), graft failure, cancer, and death, regardless of age and time on dialysis. They call attention to the need of implementation strategies to reduce the transplant waiting time and to avoid sensitization in all potential transplant candidates in order to improve the overall graft and the patient survival. For Lan et al.^13^, patients with cPRA ≥98% had a higher risk of graft loss from any cause, including death-censored allograft failure. In stratified analysis, the highest risk of graft loss among patients with cPRA ≥ 98% was observed in retransplants, but not in first transplants. There was no association between cPRA and graft loss among transplant recipients with related living donors.

A systematic review and meta-analysis compiled data from seven retrospective studies. Kidney transplantation performed in the presence of preformed donor-specific antibodies (DSAs) (n = 429) with negative flow cytometry crossmatch (FCXM) presented similar rejection rate and patient and graft survival compared to 10,677 DSA-negative transplants^14^. A positive complement-dependent cytotoxic crossmatch carries a high immunological risk, while a negative FCXM is at the lower end of the risk spectrum. Then, especially for patients who have been enlisted for a long time, the presence of a negative flow crossmatch should be taken into account, irrespective of the presence of low levels of DSAs. HLA-incompatible transplantation, including positive FCXM within a limit of channel shifts, still offers a significant survival benefit^15,16,17^.

The limitation of this study was the access only to partial data on the SNT database. We could not analyze other factors such as clinical data and the original disease. However, it was observed that female patients were more sensitized than males, as expected.

## Conclusion

Kidney transplantation is the most cost-effective renal replacement therapy for chronic kidney disease. However, the insufficient number of donors and the presence of anti-HLA antibodies act as barriers to transplant access. Our data confirm previous observations that the waiting list time is strongly affected by the degree of anti-HLA sensitization. Nonsensitized recipients had a lower risk of death, but the risk for graft loss was similar in the different cPRA groups after transplantation. This study emphasizes the need to find solutions for this group of patients that is strongly handicapped towards the access to a transplant.
